# Relationship between age and elite marathon race time in world single age records from 5 to 93 years

**DOI:** 10.1186/2052-1847-6-31

**Published:** 2014-07-31

**Authors:** Beat Knechtle, Hervé Assadi, Romuald Lepers, Thomas Rosemann, Christoph Alexander Rüst

**Affiliations:** 1 Institute of Primary Care, Zurich, Switzerland; 2 Gesundheitszentrum St. Gallen, St. Gallen, Switzerland; 3 INSERM U1093, Faculty of Sport Sciences, University of Burgundy, Dijon, France

**Keywords:** Running, Sex difference, Performance, Boys, Girls, Master runner

## Abstract

**Background:**

The aims of the study were (*i*) to investigate the relationship between elite marathon race times and age in 1-year intervals by using the world single age records in marathon running from 5 to 93 years and (*ii*) to evaluate the sex difference in elite marathon running performance with advancing age.

**Methods:**

World single age records in marathon running in 1-year intervals for women and men were analysed regarding changes across age for both men and women using linear and non-linear regression analyses for each age for women and men.

**Results:**

The relationship between elite marathon race time and age was non-linear (*i.e.* polynomial regression 4^th^ degree) for women and men. The curve was U-shaped where performance improved from 5 to ~20 years. From 5 years to ~15 years, boys and girls performed very similar. Between ~20 and ~35 years, performance was quite linear, but started to decrease at the age of ~35 years in a curvilinear manner with increasing age in both women and men. The sex difference increased non-linearly (*i.e.* polynomial regression 7^th^ degree) from 5 to ~20 years, remained unchanged at ~20 min from ~20 to ~50 years and increased thereafter. The sex difference was lowest (7.5%, 10.5 min) at the age of 49 years.

**Conclusion:**

Elite marathon race times improved from 5 to ~20 years, remained linear between ~20 and ~35 years, and started to increase at the age of ~35 years in a curvilinear manner with increasing age in both women and men. The sex difference in elite marathon race time increased non-linearly and was lowest at the age of ~49 years.

## Background

In recent years, the number of successful marathoners increased continuously. For example, in the USA, the number of successful marathon finishers increased from 25,000 in 1976 to the all-time high in 2011 with 518,000 successful finishers [1]. Recent studies investigating participation and performance trends in a large city marathon in the USA such as the ‘New York City Marathon’ showed that the increase in participants was mainly due to an increase in master runners (*i.e.* finishers of > 40 years of age) and women [2,3]. In the ‘New York City Marathon’, the number of men > 40 years increased three-fold from the 1980s to the 2000-2009, whereas the number of women increased even seven-fold [3].

Although the fastest elite marathon race times were achieved at the age of ~30 years in both female and male elite runners [4,5], it has been reported for both recreational marathoners [6] and ultra-marathoners [7] that the fastest race times can be achieved during a considerably long life span. For marathoners, the age-related loss in running performance did not occur before the age of ~50 years [6]. Mean marathons race times were nearly identical for age group runners from 20 to 49 years [6]. Also for 100-km ultra-marathoners, the fastest race times were observed during the age span of 30-49 years for men and 30-54 years for women, respectively [7].

It has been shown that race times in endurance and ultra-endurance events increased in a curvilinear manner with increasing age [3,7-11]. In these studies, data from runners older than 25 years sorted in 5-years age groups were analysed. The main findings were that running performance was maintained until the age of ~35 years followed by a moderate decrease until the age of ~50-60 years and with a sharp decline after the age of ~60 years. When the relationship was expressed between age and race times, the change was curvilinear with an increase into higher ages [6,7]. When elite and recreational athletes were compared, it seemed that the pattern of the age-related performance decline was very similar for both groups [12].

In a study by Lara *et al.*[5], the association between elite marathon race time and age in 1-year intervals from 18 to 75 years in elite women and men competing in the ‘New York City Marathon’ in 2010 and 2011 was investigated. In contrast to previous findings, the relationship between elite marathon race time and age was U-shaped [5]. The first aim of the present study was to investigate the relationship between elite marathon race times and age in 1-year intervals by using the world single age records in marathon running for each age from 5 to 93 years. A second aim of the present study was to further investigate the relationship between sex difference in elite marathon running performance and advancing age. Based upon the findings in Lara *et al.*[5], we hypothesized to confirm the U-shaped relationship between elite marathon race times and age also for world single age records in marathon running.

## Methods

### Ethics

This study was approved by the Institutional Review Board of St. Gallen, Switzerland, with a waiver of the requirement for informed consent given that the study involved the analysis of publicly available data.

### Data sampling and data analysis

The data set for this study was obtained from the website of the ‘Association of Road Racing Statisticians’ (ARRS) [13]. This website records the world single age records in marathon running in 1-year intervals from the age of 5 to 93 years for men and 5 to 92 years for women. Elite marathon race times achieved from 5 to 93 years were analysed regarding changes across age for both men and women using linear and non-linear regression analyses since the change in endurance performance and sex difference in endurance performance is assumed to be non-linear [14]. In marathons, the lowest age to officially enter the race is 18 years and we therefore started our analysis at the age of 18 years. The comparison of races times for athletes older than 80 years showed large differences in marathon race performance and we therefore performed a second analysis with race times of athletes aged 18-80 years. When the best-fit model was a non-linear (*i.e.* polynomial) regression, we compared the best-fit non-linear model to the linear model using Akaike’s Information Criteria (AICc) and F-test in order to show which model would be the most appropriate to explain the trend of the data.

## Results

For men, the fastest elite marathon race time of 2:03:23 h:min:sec was achieved by Wilson Kipsang Kiprotich, Kenia, at the age of 31 years and 198 days on September 29, 2013, in Berlin, Germany. However, Geoffrey Kiprono Mutai, Kenia, ran the fastest marathon ever on April 18, 2011, at the ‘Boston Marathon’ in a time of 2:03:02 h:min:sec. However, this time was not recognized as an official world record in marathon running by the International Association of Athletics Federations (IAAF). The course of the ‘Boston Marathon’ does not meet the criteria to be eligible for the mark since the race is a point-to-point course. For women, Paula Radcliffe, Great Britain, achieved the fastest elite marathon race time of 2:15:24.6 h:min:sec on April 13, 2003 in London, England, at the age of 29 years and 117 days. Table 1 presents the athletes who were able to achieve more than one world single age record. In men, 14 athletes reached two or more records where Ed Whitlock, Canada, achieved the highest number with 11 records. In women, 16 runners attained two or more records where Tatyana Pozdniakova, Ukrainia, holds six records.

**Table 1:** Athletes with repeated world records

**Name and origin**	**Number of records**	**Ages (years)**
**Men**		
Bucky Cox (USA)	2	5, 6
Zhu-hong Li (CHN)	2	16, 17
Feyisa Lelisa Gemechu (ETH)	2	20, 22
Wilson Kipsang Kiprotich (KEN)	2	29, 31
Emanuel Mutai (KEN)	2	32, 35
Jaouad Gharib (MAR)	2	36, 39
Jackson Kipngok Yegon (KEN)	2	45, 47
Clive Davies (USA)	2	64, 66
Ed Benham (USA)	2	77, 84
Mike Fremont (USA)	2	88, 90
Fauja Singh (ENG)	3	91-93
Wesley Paul (USA)	4	7, 9, 11, 12
Yoshihisa Hosaka (JPN)	4	59-61, 63
Piet vanAlphen (NED)	5	51-55
Ed Whitlock (CAN)	11	68-70, 72-76, 80-82
**Women**		
Julie Mullin (USA)	2	9, 10
Birhane Dibaba Adugna (ETH)	2	19, 20
Aselefech Mergia (ETH)	2	21, 23
Jung-Ok Kim (KOR)	2	56, 57
Angela Copson (ENG)	2	62, 66
Emmi Lüthi (SUI)	2	63, 65
Margaret Davis (USA)	2	83, 86
Jennifer Amyx (USA)	3	5-7
Paula Radcliffe (ENG)	3	28, 29, 31
Gwen McFarlan (CAN)	3	74, 76, 80
Ida Mintz (USA)	3	78, 79, 84
Irina Mikitenko (GER)	4	36, 39-41
Helga Miketta (GER)	4	67, 70-72
Betty-Jean McHugh (CAN)	4	75, 81, 82, 85
Mavis Lindgren (USA)	4	87-90
Tatyana Pozdniakova (UKR)	6	43, 46, 47, 49-51

### Relationship between elite marathon race time and age

**Figure 1:**
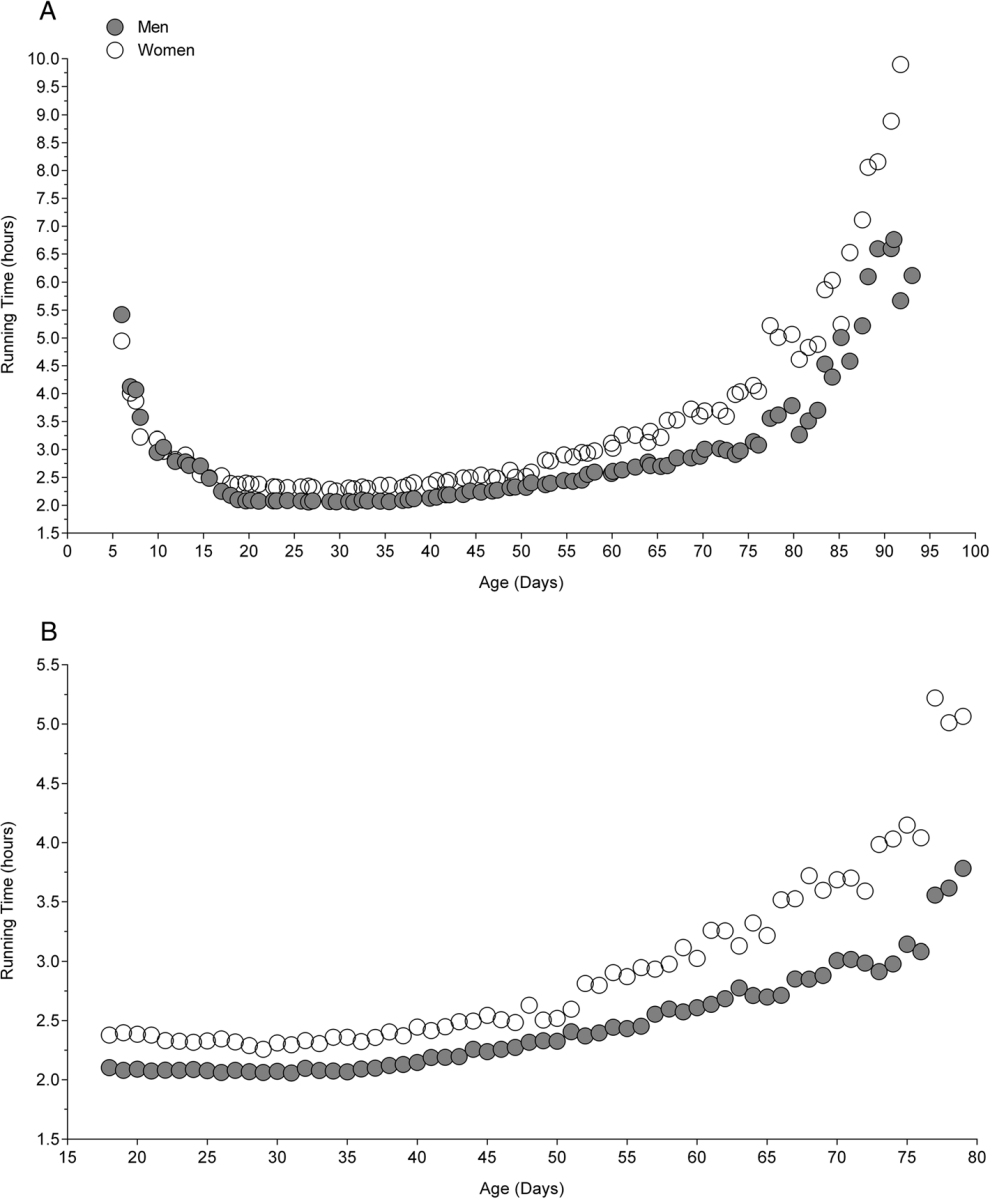
Relationship between marathon race time and age for the world single age records in women and men from 5 to 93 years (Panel A) and from 18 to 80 years (Panel B).

Figure 1 presents the relationship between elite marathon race time and age for women and men from 5 to 93 years (Figure 1A) and from 18 to 80 years (Figure 1B). From 5 to 93 years, the relationship was non-linear for both women and men (*i.e.* polynomial regression 4^th^ degree). Also for 18-80 years, the relationship was non-linear (*i.e.* polynomial regression 5^th^ degree) (Table 2). Regarding the group 5 to 93 years (Figure 1A), the curve was U-shaped where performance improved from 5 to ~20 years. From 5 years to ~15 years, boys and girls performed very similar. Between ~20 and ~35 years, performance was very linear (Figure 1A and B), but started to increase at the age of ~35 years in a curvilinear manner for both men and women with increasing age in both women and men.

**Table 2:** Equations of the non-linear regressions for running times and sex differences

		**Equation**	**SE**	**r**	**r**^**2**^
Running time	Men 5-93 years	y = 6.65 - 0.0012 · x + 0.00000 · *x*^2^ - 0.000000 · x^3^ + 0.00000 · x^4^	0.28	0.97	0.94
	Women 5-92 years	y = 6.41 - 0.0012 · x + 0.00000 · *x*^2^ - 0.000000 · x^3^ + 0.00000 · x^4^	0.26	0.98	0.97
	Men 18-80 years	y = - 1.95 + 0.0015 · x - 0.00000 · *x*^2^ + 0.000000 · x^3^ - 0.00000 · x^4^ + 0.000000 · x^5^	0.05	0.99	0.98
	Women 18-80 years	y = - 3.37 + 0.0022 · x - 0.00000 · *x*^2^ + 0.000000 · x^3^ - 0.00000 · x^4^ + 0.000000 · x^5^	0.12	0.98	0.97
Sex difference	5-92 years	y = - 183.23 + 0.12 · x - 0.000032 · *x*^2^ + 0.000000 · x^3^ - 0.00000 · x^4^ + 0.000000 · x^5^ - 0.00000 · x^6^ + 0.000000 · x^7^	14.73	0.93	0.87
	18-80 years	y = 73.52 - 0.015 · x + 0.000002 · *x*^2^ - 0.000000 · x^3^ + 0.00000 · x^4^	6.45	0.94	0.89

### Relationship between sex difference and age

**Figure 2:**
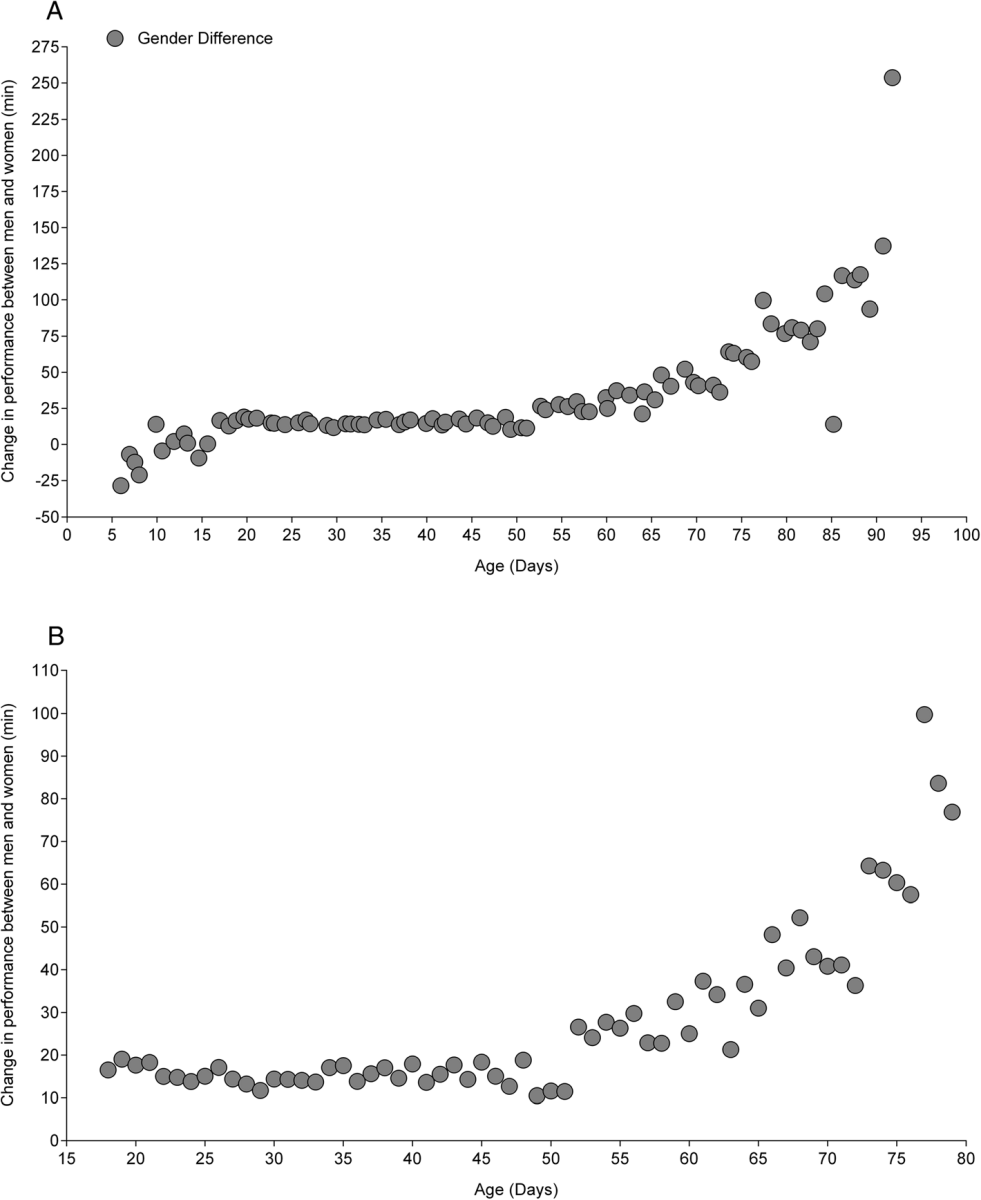
Relationship between sex difference and age from 5 to 93 years (Panel A) and from 18 to 80 years (Panel B).

Figure 2 presents the relationship between sex difference and age from 5 to 93 years (Figure 2A) and from 18 to 80 years (Figure 2B). In contrast to the relationship for elite marathon race time and age as an U-shaped curve, sex difference increased in 5 to 93 years (Figure 2A) non-linearly (*i.e.* non-linear polynomial regression 7^th^ degree) from 5 to ~20 years, remained unchanged at ~20 min from ~20 to ~50 years and increased thereafter. In 18-80 years, the sex difference remained unchanged at ~20 min from ~20 to ~50 years and increased thereafter (*i.e.* non-linear polynomial regression 4^th^ degree, Figure 2B). The sex difference was lowest at the age of 49 years (*i.e.* 7.5%, 10.5 min).

## Discussion

The first aim of this investigation was to analyse the relationship between elite marathon race times and age when including the whole range of ages and by using an analysis with 1-year intervals for the world single age records. The second aim was to evaluate the sex difference in running performance with advancing age.

### Relationship between elite marathon race time and age

The most important finding was that the association between elite marathon race times and age was curvilinear for both elite women and men for 5-93 years and 18-80 years. Elite marathon race times showed a linear progress from 18-35 years and started to increase in a curvilinear manner at the age of ~35 years. We hypothesized confirming the U-shaped relationship between elite marathon race times and age based upon the findings in Lara *et al.*[5]. However, the relationship between elite marathon race times and age was again a curvilinear association as has been reported for marathoners and ultra-marathoners when investigating age group marathoners and ultra-marathoners sorted in 5-year [3,7,8] or 10-year intervals [6] between 20 and 79 years.

Even in recreational marathoners, no differences in elite marathon race times of runners aged from 20 to 55 years were found [6,15]. These studies showed that marathon running times increased exponentially with increasing age starting at the age of ~35 years. Consequently, performance decreased with increasing age. Performance decreased after the age of ~50 years where the decrease became dramatic after the age of ~80 years. The decrease in endurance performance is mainly due to the decrease in maximum oxygen uptake (VO_2_max). VO_2_max decreases with age and is a factor in slower times with increasing age. The decline in VO_2_max with age appears to be inevitable [16]. VO_2_max declines by ~10% per decade in both women and men regardless of the activity level [17]. However, high-intensity exercise may reduce this decrease by ~50% in young and middle-aged men, but not older men [17]. Middle-aged and older women do not appear to be able to reduce loss rates in VO_2_max to less than 10% per decade [17].

It has been shown by Lepers and Cattagni [3] that elite marathon race times started to increase at the age of ~35 years in both women and men. In contrast to existing reports and the findings in the present study, Lara *et al.*[5] found an U-shaped relationship between elite marathon race times and age where the fastest race times were achieved at the age of 27 years in men and 29 years in women. A potential explanation for the different findings could be the different samples of athletes and the larger sample in Lara *et al.*[5] including 20 participants for each age while we included only one athlete per age.

An interesting observation was that fact that from 5 years to ~15 years, boys and girls performed very similar. After the age of ~15 years, male adolescents started to run faster than female adolescents. Before puberty, body dimensions are very similar in both boys and girls and boys and girls are only different in having different genitalia (sex organs). With puberty, body characteristics such as bone length, fat mass and muscle mass start to change [18]. With the start of puberty, testosterone starts to increase in boys leading to an increase in skeletal muscle mass whereas fat mass increases in girls [19]. Due to the higher muscle mass strength is higher in boys compared to girls [20,21]. Additionally, aerobic capacity will become higher in boys compared to girls [22] and endurance performance will be higher in body than in girls [23,24].

### Relationship between sex difference and age

The second important finding was that the sex difference in elite marathon race time was a U-shaped and was lowest at the age of ~49 years. In contrast, Lara *et al.*[5] found a stable sex difference of ~18.7 ± 3.1% from 18 to 57 years and the lowest sex difference of 10.2 ± 5.5% was obtained at the age of 29 years. The difference between the findings in Lara *et al.*[5] and our findings might be explained by the different performance levels of the investigated subjects and the number of investigated subjects. An interesting finding was that the sex difference was lowest at the age of ~49 years and was higher in younger and older ages than ~49 years although the elite marathon race times showed a rather linear progress from 18 to 35 years. Normal aging in humans is associated with a progressive decrease in skeletal muscle mass [25,26] and strength [27,28]. A gradual loss of muscle fibres starts at the age of ~50 years and continues such that by the age of ~80 years, ~50% of the fibres are lost from the limb muscles [29].

There seemed to be differences between the sexes regarding this age-related loss in skeletal muscle mass. In 68-78 years old women and men, the rate of loss in leg muscle was significantly higher in men than in women [30] and the prevalence of sarcopenia was ~31% in women and ~53% in men older than 80 years [25]. There seemed also to be differences between the sexes regarding the anthropometric predictors of physical performance in older women and men [27] and strength of the lower limb [31,32]. In subjects at the age of ~73 years, the quality of the leg muscles was related to chair rise time and gait speed in men, but not in women [27]. In men, the muscle quality is more important to functional performance than in women and maintaining high quality skeletal muscle is particularly important for older men [27].

### Limitations

This study is limited due to the lack of inclusion of physiological variables (*i.e.* VO_2_max, lactate threshold, running economy), training characteristics (*i.e.* running speed during training, training volume), previous experience and nationality. A further limitation is that the statistical analysis was performed only by including the world single age records for women and men. It would be interesting to study the relationship between marathon race and age in a larger set of marathoners. The results listed in the ‘Association of Road Racing Statisticians’ [13] are not necessarily the best performances ever accomplished. They list the fastest performances for each single age and for each of the standard distances. Performances are subject to the same standards as listing for national records plus the additional requirement that the runner's date of birth as well as the race date must be known. These are required to be able to document the runner's exact age at the time of the performance. Single age records meeting the qualifying standards may be expected to be fairly reliable. At older and younger ages, the best times known are listed. A further important limitation of the study is that the data are cross-sectional. With longitudinal data, different results could have been observed.

## Conclusions

Elite marathon race times improved from 5 to ~20 years, remained linear between ~20 and ~35 years, and started to increase at the age of ~35 years in a curvilinear manner with increasing age in both women and men. The sex difference in elite marathon race time increased non-linearly and was lowest at the age of ~49 years. Future studies need to confirm these findings in a large data set.

## Competing interests

The authors declare no competing interests.

## Authors’ contributions

BK drafted the manuscript, RL and HA conceived the study, CR and RL performed the statistical analyses, TR helped in drafting the manuscript. All authors read and approved the final manuscript.

## Pre-publication history

The pre-publication history for this paper can be accessed here:

http://www.biomedcentral.com/2052-1847/6/31/prepub
